# The Effect of the Small Indian Mongoose (*Urva auropunctatus*), Island Quality and Habitat on the Distribution of Native and Endemic Birds on Small Islands within Fiji

**DOI:** 10.1371/journal.pone.0053842

**Published:** 2013-01-17

**Authors:** Craig G. Morley, Linton Winder

**Affiliations:** 1 School of Forestry and Primary Industries, Waiariki Institute of Technology, Rotorua, New Zealand; 2 Department of Natural Sciences, Faculty of Social and Health Sciences, Unitec Institute of Technology, Auckland, New Zealand; Liverpool John Moores University, United Kingdom

## Abstract

This study investigated the effect of the presence of introduced mongoose, environmental quality and habitat on the distribution of native and endemic birds on 16 small islands within Fiji. In total, 9055 birds representing 45 species were observed within four key habitats (forest, villages, crop land and coastal vegetation) on the 16 islands, half of which had mongoose present. Previous studies attribute bird declines and extirpation anecdotally to the mongoose. The presence of mongoose, environmental quality and habitat type had a measurable influence on observed extant native and endemic bird communities. We conclude that three ground birds; *Gallirallus phillipensis*, *Anas supericiliosa* and *Porphyrio porhyrio* were negatively influenced by the presence of mongoose and that *Ptilinopus perousii, Phigys solitarius, Chrysoenas victor, Ducula latrans, Clytorhyrchus vitiensis, Pachycephala pectoralis, Prospeia tabunesis*, and *Foulehaio carunculata* were particularly dependent on good quality forest habitat. Conservation priorities in relation to protecting Fiji's endemic birds from the effect of mongoose are discussed and preventative measures suggested.

## Introduction

Since AD1500 it is estimated that at least 153 bird species have become extinct worldwide, 92% of them being endemic to islands. Currently, about 1200 species are considered globally threatened [1]. Bird extinctions have been attributed to a number of anthropogenic causes (e.g. overexploitation, habitat fragmentation and habitat destruction) including the introduction of invasive species [2]–[5].

Fiji currently has 57 extant breeding landbirds, of which 46% are endemic [6]–[7]. Several additional species from the Fiji Islands comprising a duck (*Dendrocygna arcuata*), and an owl (*Tyto longimembris*) were last seen in the late 1800s and are considered extinct [8]. Other birds such as the red-throated lorikeet (*Charmosyna amabilis*) have not been observed for over a decade [9] and species such as the pink-billed parrotfinch (*Erythrura kleinschmidti*) and long-legged warbler (*Trichocichla rufa*) are rarely seen and are thought to be in serious decline [7]. In addition, a grebe (*Tachybaptus novaehollandiae*), four megapodes (*Megapodius alimentum*, *M. amissus*, *M. amissus/molistructor* and *Megavitiornis altirostris*) and two pigeons (*Ducula lakeba* and *Natunaornis gigoura*) are only known from subfossils [10]–[11], and seven extinct rails from seven sympatric genera were present in the immediate pre-human period on Viti Levu [12].

It is known that the introduction of invasive species has had a profound effect on island ecosystems and they are a key reason why many extant native bird species are in decline [5], [13]–[16]. Over time, invasive species can erode the biological foundations of an ecosystem, causing considerable and irreparable damage. By the time wildlife managers realise that action is necessary, much damage has already been done with the consequent loss of vulnerable island species [17]. However, conservation measures to reduce habitat destruction are often implemented whilst the impact of invasive species are overlooked by land managers, politicians, and local communities.

It is recognised that the ecological status of the Fiji Islands has declined since the arrival of people and the consequent introduction of feral mammals such as rats (*Rattus* sp.), cats (*Felis catus*), and dogs (*Canis lupus familiaris*). The focus of this study, the small Indian mongoose (*Urva auropunctatus* synonyms *Herpestes auropunctatus*, and *H. javanicus*; hereafter called mongoose) was introduced to the Fiji Islands in 1883 [18]–[20]. Mongoose have been implicated anecdotally in the decline of many of Fiji's birds such as the barred-wing rail, *Nesoclopeus poecilopterus* [21], the Pacific black duck, *Anas superciliosa* [22], the banded rail, *Gallirallus philippensis* [23], the purple swamphen, *Porphyrio porhyrio* [24] and friendly ground dove, *Gallicolumba stairi* [7]. Mongoose are also considered to have caused avifauna declines on other islands including: the nene (*Branta* [*Nesochen*] *sandvicensis*) in Hawai'i [25], the two quail doves (*Oreopeleia mystacea* and *Geotrygon mystacea*) in the Caribbean [26], [27] and Audubon's shearwater (*Puffinus l'herminierri*) on Mauritius [28].

Despite such reports associating mongoose with bird extinctions, there are no documented cases with substantive evidence that imply causation. Bird census data is often not available prior to such introductions [29] which makes it difficult to causatively relate declines to the mongoose. Additionally, some studies found the mongoose to be neither harmful nor beneficial [30].

Most landbird studies in Fiji have been undertaken on the four main islands of Viti Levu, Vanua Levu, Taveuni, and Kadavu, and have generally concluded that problems were created by the introduction of invasive species [31]–[36]. Although some bird work has been done on Fiji's outer islands the majority was completed over 80 years ago [37]–[43] or remains unpublished [44]. Yet these islands have populations of native species that are particularly vulnerable to extinction. Although there is an awareness of the likely problems of invasive species on bird populations within the Fiji Islands at governmental and local levels, no studies have been undertaken to quantify or investigate this impact systematically. The primary objective of this study was to compare the bird communities of islands known to have mongoose populations present to those without. We hypothesised that there would be measurable differences in bird communities between the two sets of islands. Fiji is an ideal location for such a study, as the multitude of smaller islands provides a means to robustly test this hypothesis.

## Materials and Methods

### Ethics Statement

The University of the South Pacific's Research Committee, responsible for ethics assessment, approved this study. All necessary permits were obtained for the described field studies through full consultation with the Roko Tui.

### Study Area

The Fiji Islands are south of the equator and north of the Tropic of Capricorn, proximal to the 180° meridian and lie on the Fiji Platform and the Lau Ridge of the Indo-Australian Plate [45]. The country's territorial limits cover 1.3 million km^2^, but only 18,333 km^2^ is land [46]. There are more than 300 islands in the group with two main islands; Viti Levu (10,390 km^2^: elevation 1,323 m) and Vanua Levu (5,535 km^2^: elevation 1,032 m). The islands are made up of a variety of Eocene to Late Miocene plutonic, volcanic and sedimentary rocks [46]. Fiji has nine principal vegetation types; lowland rainforest, upland rainforest, cloud forest, mangrove forest and scrub, plant communities degraded by fire (*talasiga*), coastal strand vegetation, freshwater wetland vegetation and smaller island vegetation [47]. The latter refers to lowland rain forests where the species richness of the canopy trees has been highly modified by people [43].

**Figure 1:**
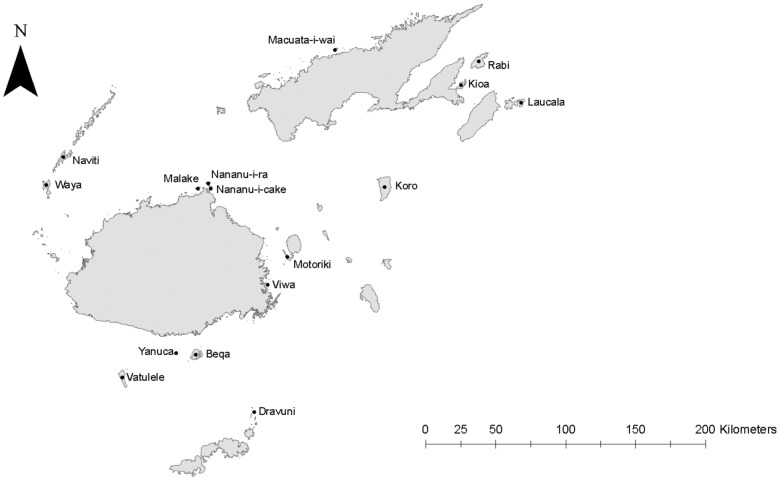
Map of Fiji with islands surveyed during the study identified.

This study was carried out on 16 small offshore islands in the Fiji group selected *a priori* where half of the islands had known mongoose populations (Figure 1). *A priori* categorisation was confirmed by setting 40–60 mongoose traps (at 200 m intervals) on each island for an eight day period during the study. Island selection was supported by extensive preliminary research [7], [21], [34] and consultation with various authorities within Fiji in order to pair, as far as was possible, the eight islands with known mongoose populations with comparable mongoose-free islands by consideration of island size (km^2^), elevation (m), and habitat quality (Table 1). Habitat quality represented the impact of human settlement and the presence of remaining primary forest. Island quality was scored by the investigator when each island was visited on a 1–10 interval-based scale. A score of 1 represented the poorest quality, where the island habitat was highly modified with exotic species dominating the vegetation. A score of 10 represented excellent quality, where there was relatively little evidence of anthropogenic habitat disturbance with significant tracts of intact primary forest remaining.

**Table 1:** Environmental variables used in the study recorded for 16 islands.

Island	Size (km^2^)	Elevation (m)	Island quality	Island-wide endemic, native, and introduced bird species richness	Number of mongoose trapped
Vatulele^1^	31.3	33	7	2, 19, 4	0
Naviti^2^	34	338	3	5, 9, 0	0
Laucala^3^	12.2	265	9	11, 14, 5	0
Waya^4^	22	502	3	6, 10, 3	0
Moturiki^5^	10.9	132	7	8, 14, 3	0
Dravuni^6^	0.8	111	2	2, 8, 1	0
Koro^7^	104	561	8	9, 13, 2	0
Viwa^8^	0.6	49	7	7, 12, 4	0
Beqa^1^	36.2	439	3	6, 9, 5	78
Kioa^2^	18.6	305	9	10, 15, 3	23
Macuata-i-wai^3^	3	184	1	5, 14, 3	78
Malake^4^	4.5	219	1	3, 11, 5	43
Nananu-i-cake^5^	3	73	3	6, 11, 4	13
Nananu-i-ra^6^	2.7	73	2	6, 10, 4	13
Rabi^7^	68.8	463	6	8, 12, 4	46
Yanuca^8^	1.5	137	1	4, 3, 4	45

Superscripts indicate island pairs selected *a priori*, half of which had known mongoose populations. Mongoose trapping was done to confirm *a priori* island selection. Island quality represented the effects of human-induced habitat change and was scored on a 1–10 interval scale; 1 being ‘poor’ (severe impact) and 10 being ‘excellent’ (relatively little impact).

### Field Surveys

Randomised bird point counts were done within four habitats on each island between February 2002 and May 2003. Habitats selected represented the predominant vegetation types evident within these islands which were all modified to some extent by human activity. The four habitats were: (i) village – areas that consisted of dwellings often next to the foreshore, with open grassland areas, ornamental plants and trees such as *Hibiscus rosa-sinensis*, *Plumeria rubra*, *Zingiber officinale*, *Alpinia purpurata*, *Ixora* spp. *Bougainvillea spectabilis*, and *Delonix regia*; (ii) crop areas or talasiga - highly modified areas with crop plantations, herbaceous and shrubby communities such as *Colocasia* spp., *Manihot esculenta*, *Artocarpus altilis*, *A. integra*, *Musa* spp., *Carica papaya*, *Mangifera indica*, *Annona muricata*, with the common invasives *Lantana camara* and *Mimosa pudica* present; (iii) relatively intact primary forest with trees such as *Myristica* c.f. *gillespieana, Canarium harveyi*, *Fiscus oblique*, *Endospermum macrophyllum, Incarpus fagifer*, and *Sterculia vitiensis* (intact primary forest was rare on the smaller islands and non-native species such as *Samanea saman, Casuarina equisetifolia*, *Spathodea campanulata*, and *Leucaena leucoceophala* dominated the forest community); (iv) coastal scrub - a distinctive flora with *Rhizophora samoensis* and *R. stylosa, Bruguiera gymnorhiza*, *Cocos nucifera*, *Pandanus* spp. *Barringtonia asiatica*, *Terminalia catappa*, *Passiflora foetida* and *Ipomoea pes-caprae* present.

One point count station was established within each of the four habitats on each island. Stations were located at random within each habitat type using a base map following a reconnaissance trip. Each point count station was at least 250 m away from any other station and some allowance on location was made in order to provide suitable and comparable vantage points. The observer remained stationary at each point count station whilst surveying; birds were recorded when they were heard calling, when they were observed flying directly overhead or when perched. Distance bands (no greater than 50 m) and directions were used to ensure that each bird was counted only once. Surveying was done on five separate days at each station and data recorded from these five counts were combined to provide a single record for each station. Each 15 minute count was conducted at a time between 0600 and 0900, and was commenced five minutes after the arrival of the observer to minimise the effects of disturbance. Counts were conducted on consecutive days unless it rained when the count was postponed to the next fine day. All species were recorded, but for the following analyses, introduced birds, seabirds, and singletons were excluded.

### Data Analysis

#### (i) The presence of mongoose and island quality

For each island, a record of species was generated by pooling observations from the four habitats surveyed in order to represent the island-wide species assemblage. An assemblage was defined as the species present that comprised the community at a given location. Abundances were not pooled as they did not represent a meaningful island-wide estimate [48]. The effect of island quality and presence of mongoose on richness was determined using GLM (SPSS version 19).

Partial constrained ordination [49] was used to investigate whether bird community assemblages were dependent on either the presence of mongoose or island quality using the partial redundancy analysis (pRDA) routine of Canoco Version 4 [50]. This technique is an analogue of multiple regression and allows community variation to be explained by external environmental variables. The method compares a dependent matrix (in this case species assemblage) to explanatory variables (in this case environmental gradients that recorded island size, island quality, etc.). The method allows inclusion of explanatory variables that are of primary interest (island quality or the presence of mongoose) and covariables which are other explanatory variables of lesser interest (island size, etc.) that have a known or hypothesized effect on the variation within the dependent matrix [50]. The influence of either island quality or mongoose presence may then be measured beyond the influence of other covariables included within the model. The analysis determines the influence of the selected environmental gradients on species assemblage. Significance testing may then be conducted to determine whether these explanatory variables measurably influenced species assemblage.

Preliminary analyses established that the linear method was the most appropriate form of analysis and that island size and elevation should be included as covariables. Firstly, the effect of the presence of mongoose on island-wide species assemblage was tested with mongoose presence/absence as the explanatory variable, and island quality, log_10_ island size and log_10_ elevation as covariables. The significance of the first ordination axis (that represented in this case mongoose presence/absence) was tested using a Monte Carlo permutation test. Secondly, the effect of the environmental quality of the island was tested using the explanatory variable island quality, with mongoose presence/absence, log_10_ island size and log_10_ elevation as covariables. The significance of the first ordination axis (that represented in this case island quality) was again tested using a Monte Carlo permutation test. We therefore removed variability caused by known covariables prior to determining whether either island quality or mongoose presence/absence independently influenced species assemblage. Species-environment biplots were produced for each analysis, and we displayed species that were measurably dependent upon the explanatory variable being tested (species not displayed were therefore not influenced by the explanatory variable). Species that were measurably influenced by the explanatory variable being tested were displayed by setting an appropriate inclusion rule threshold, equivalent to the percentage variation explained by Axis 1 of the ordination in CanoDraw [49].

#### (ii) Within-islands habitats

Firstly, in order to compare habitats, rarefraction curves were generated in Biodiversity Pro (Version 2) following the method of Hurlbert [51]. Rarefraction controlled for differing sample sizes, which was expected as detectability inevitably varied between habitats. A qualitative comparison of rarefraction curves was undertaken to compare between-habitat richness; a formal comparison was not done because some samples were small (15 individuals) which would have led to convergence if sample sizes were equalised [47], [52].

Secondly, an analysis was done that compared community assemblage for each of the four surveyed habitats. For each habitat separately, the effect of island quality and presence of mongoose on richness was determined using GLM and partial constrained ordination (pRDA) was used to investigate whether species assemblage was dependent on either the presence of mongoose or island quality. In the pRDA analysis log_10_(n+1) transformed counts were used. Each habitat type was included, in turn, as the explanatory variable, with mongoose presence/absence, island quality, log_10_ island size and log_10_ altitude set as covariables in a similar manner to the analysis above. Species-environment biplots were produced for each analysis, and only species whose variability were explained by the ordination axis being tested were included as explained above.

## Results

It was confirmed that the *a priori* selection of islands into those with and without mongoose was robust. No mongoose were recorded on islands designated as mongoose-free, whilst mongoose were recorded on all the other islands (Table 1). Island quality varied due to the level of disturbance caused by human activity and spanned a range from poor to very good for islands either with or without mongoose (Table 1). Laucala and Kioa were considered to be in the best condition, whilst Dravuni, Malake, Nananu-i-ra, Macuata-i-wai, and Yanuca were in relatively poor condition.

In total, 9,055 bird sightings of 45 species were made on the 16 islands. The seabirds *Butorides striatus*, *Fregata ariel, Pluvialis fulva*, *Sula sula* and the singletons *Cacomantis pyrrophanus*, *Falco peregrinus, Gallicolumba stairi* and *Tyto alba* were recorded; the number of bird sightings totalled 9027 excluding these species (Tables 1 and 2; Tables S1 and S2). Of the remaining 37 species recorded, 11 (30%) were endemic, 19 (51%) were native and 7 (19%) were introduced (Table 2). Numerically, introduced species were the most abundant and as expected high numbers of introduced birds were observed in the vicinity of the villages whilst few were observed in forested areas.

**Table 2:** Summary of bird species recorded during the study (excluding seabirds and singletons).

Family	Species	Type	Code	Feeding and habitat preferences
Alcedinidae	*Todirhamphus chloris*	N		Insectivore + lizards, birds and crabs. Any habitat.
Anseriformes	*Anas supericiliosa*	N	A. supe	Seeds and aquatic plants. Wetlands
Apodidae	*Aerodramus spodiopygia*	N		Insectivore. Cliffs, caves and open areas.
Ardeidae	*Egretta sacra*	N	E. sacr	Fish, worms and crustaceans. Coastal Forest.
Artamidae	*Artamus mentalis*	E		Insectivore. Any habitat, mainly open areas.
Campephagidae	*Lalage maculosa*	N	L. macu	Insects and fruit. Any habitat.
Columbididae	*Chrysoenas luteovirens*	E		Frugivore. Mature forest and forest patches.
Columbididae	*Chrysoenas victor*	E	C. vict	Frugivore. Mature forest and forest patches.
Columbididae	*Columba vitiensis*	N	Co. viti	Fruits and berries. Disturbed forest.
Columbididae	*Ducula latrans*	E	D. latr	Frugivore. Mature Forest.
Columbididae	*Ducula pacifica*	N		Frugivore. Coastal Forest.
Columbididae	*Ptilinopus perousii*	N	P. pero	Frugivore. Mature Forest and forest patches.
Columbididae	*Ptilinopus porphyraceus*	N		Frugivore. Found low in trees and shrubs, under canopy.
Columbididae	*Streptophella chinensis*	I		Grains. Open woodland and/or agricultural areas.
Cracticidae	*Gymnorhina tibicen*	I		Omnivore. Lowlands and coconut plantations.
Falconiformes	*Accipter rufitorques*	E		Bird of prey. Open woodland and/or agricultural areas.
Falconiformes	*Circus approximans*	N		Bird of prey. Open woodland/agricultural areas/forest edges.
Hirundinidae	*Hirundo tahitica*	N	H. tahi	Insectivore. Coastal Forest.
Meliphagidae	*Foulehaio carunculata*	N	F. caru	Insects and nectar. Any habitat (Taveuni only).
Meliphagidae	*Myzomela jugularis*	E		Nectivore. Any habitat - wherever there are flowering trees
Monarchidae	*Clytorhyrchus vitiensis*	E	Cl. viti	Insects and fruit. Forest and thick scrub.
Monarchidae	*Mayrornis lessoni*	E	M. less	Insectivore. Forest and suburban gardens.
Monarchidae	*Myiagra vanikorensis*	N		Insectivore. Any habitat.
Monarchidae	*Rhipidura spilodera*	N	R. spil	Insectivore. Forest and well-wooded areas.
Pachycephalidae	*Pachycephala pectoralis*	N	P. pect	Insects and fruit. Mature Forest.
Phasianidae	*Gallus gallus*	I		Omnivore. Secondary vegetation, forests, and wetlands.
Ploceidae	*Erythrura pealii*	E	E. peal	Seeds and insects. Open woodland and/or agricultural areas.
Ploceidae	*Amandava amandava*	I		Graminivorous. Open woodland and/or agricultural areas.
Psittacidae	*Phigys solitarius*	E	P. soli	Nectivore and fruit. Any Habitat.
Psittacidae	*Prospeia tabunesis*	E	P. tabu	Fruits, seeds and flowers. Mature Forest and forest patches.
Pycnonotidae	*Pycnonotus cafer*	I		Omnivore. Open woodland and/or agricultural areas.
Rallidae	*Gallirallus philippensis*	N	G. phil	Omnivore. Secondary vegetation, forests, and wetlands.
Rallidae	*Porphyrio porhyrio*	N	P. porh	Omnivore. Secondary vegetation, forests, and wetlands.
Sturnidae	*Aplonis tabuensis*	N	A. tabu	Nectivore and fruit. Any habitat.
Sturnidae	*Acridotheres fuscus*	I		Fruit, seeds and insects. Open woodland and/or agricultural areas.
Sturnidae	*Acridotheres tristis*	I		Fruit, seeds and insects. Open woodland and/or agricultural areas.
Zosteropidae	*Zosterops lateralis*	N		Omnivore. Mature forest and forest patches.

Type denotes that the species is considered to be native (N), endemic (E) or introduced (I). Code represents the abbreviations used in pRDA biplots (Figures 3, 4, 7, 8). Brief feeding and habitat preferences for birds observed during the study are also given.

**Figure 2:**
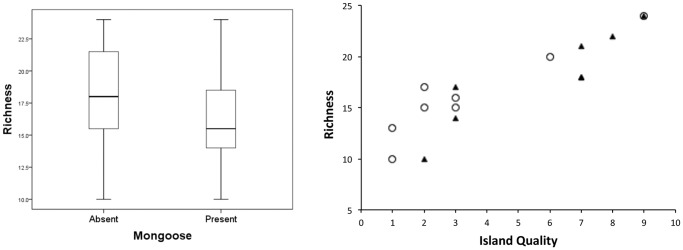
Island-wide species richness recorded during the study. Diagram a) represents richness as box plots on islands in relation to the presence or absence of mongoose; diagram b) represents richness in relation to island quality for islands where mongoose were absent (triangle) or present (open circle).

All eleven endemic species were observed on Laucala, with ten on Kioa. Nine endemic species were recorded on Koro, with the exceptions of the golden and orange dove, which have never been recorded on this island [7]. Pan-Pacific species were found widely. Richness on mongoose-absent islands appeared slightly higher than those with mongoose (Figure 2), but GLM revealed no measurable effect (F = 3.2, P = 0.1, d.f. = 1,13; Figure 2). Richness was strongly positively influenced by island quality (F = 69.8, P<0.001, d.f. = 1, 13; Figure 2).

**Figure 3:**
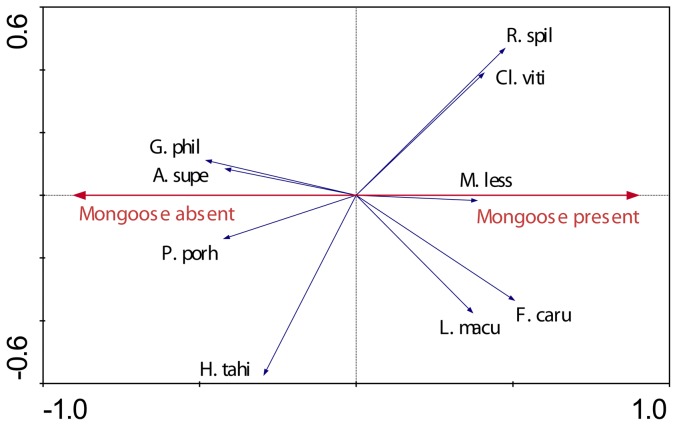
Species-environment biplot from pRDA summarizing island-wide differences in bird assemblages attributed to the presence or absence of mongoose. The diagram includes species that are measurably influenced by Axis 1 (in this case presence/absence of mongoose); arrows indicate directionality of relationship. Species with arrows that are approximately parallel to explanatory axis are more strongly influenced by the variable.

**Figure 4:**
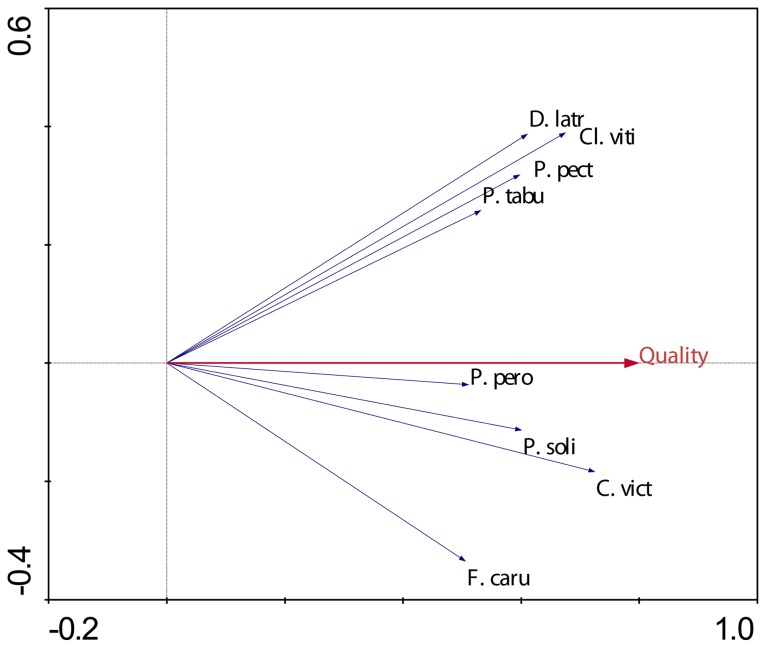
Species-environment biplot from pRDA summarizing island-wide differences in bird assemblages attributed to island quality. Refer to Fig. 3 for more details.

pRDA revealed that the presence or absence of mongoose had a weak but measurable influence on species assemblage, explaining 8.4% of the observed variation (F = 1.746, P = 0.048). The biplot revealed that three ground-active species (*G. phillipensis*, *A. supericiliosa* and *P. porhyrio*) were strongly dissociated with the presence of mongoose (Figure 3) whilst some species had an apparent positive association with mongoose. Island quality (Figure 4) had a strong effect on species assemblage, explaining 17.8% of the observed variability (F = 3.711, P = 0.002). The biplot revealed that eight species were measurably positively associated with island quality: *Ptilinopus perousii, Phigys solitarius, Chrysoenas victor*, *Ducula latrans, Clytorhyrchus vitiensis, Pachycephala pectoralis, Prospeia tabunesis*, and *Foulehaio carunculata*.

**Figure 5:**
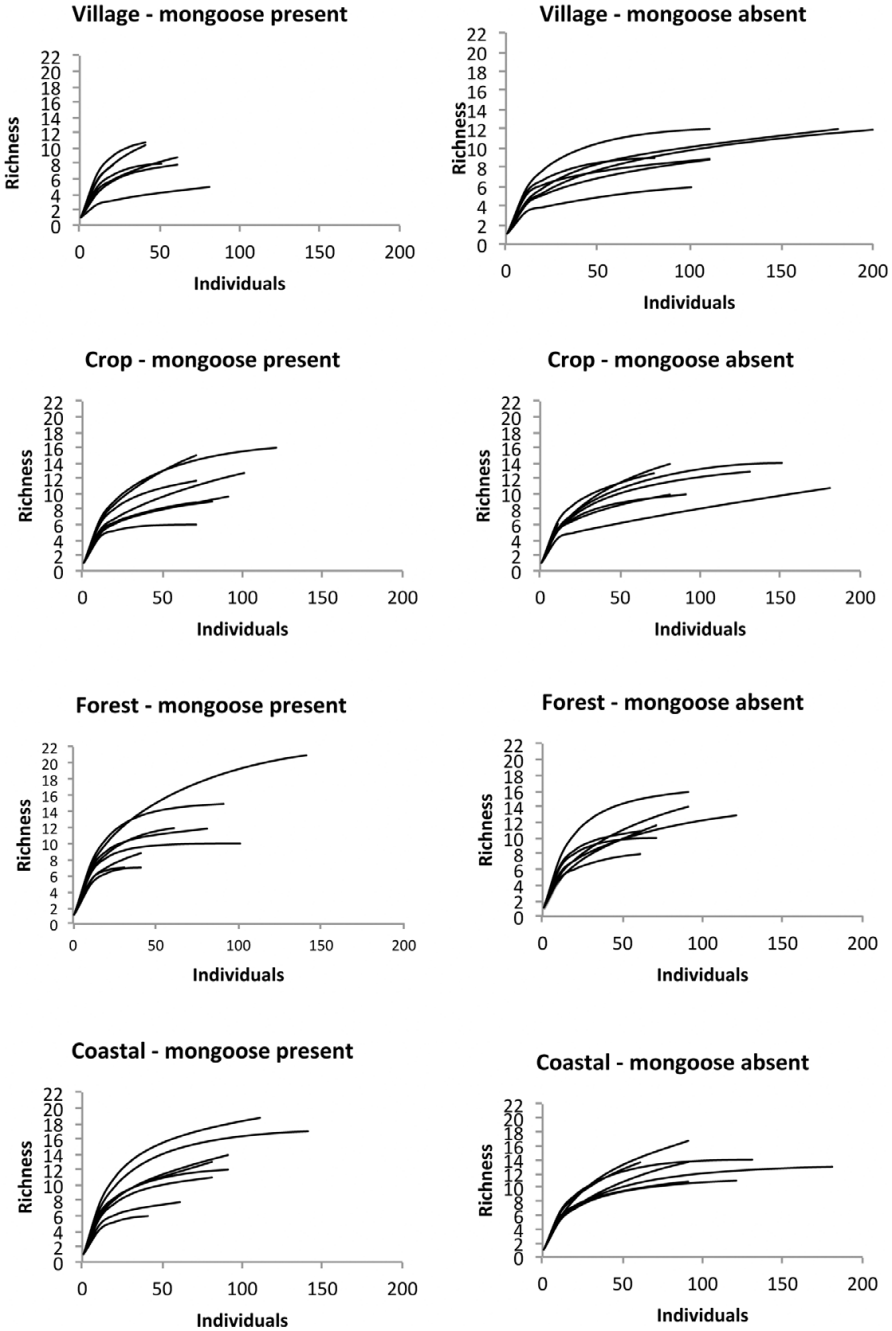
Rarefraction curves showing accumulation of species with increasing sample size for each of the four habitats sampled.

**Figure 6:**
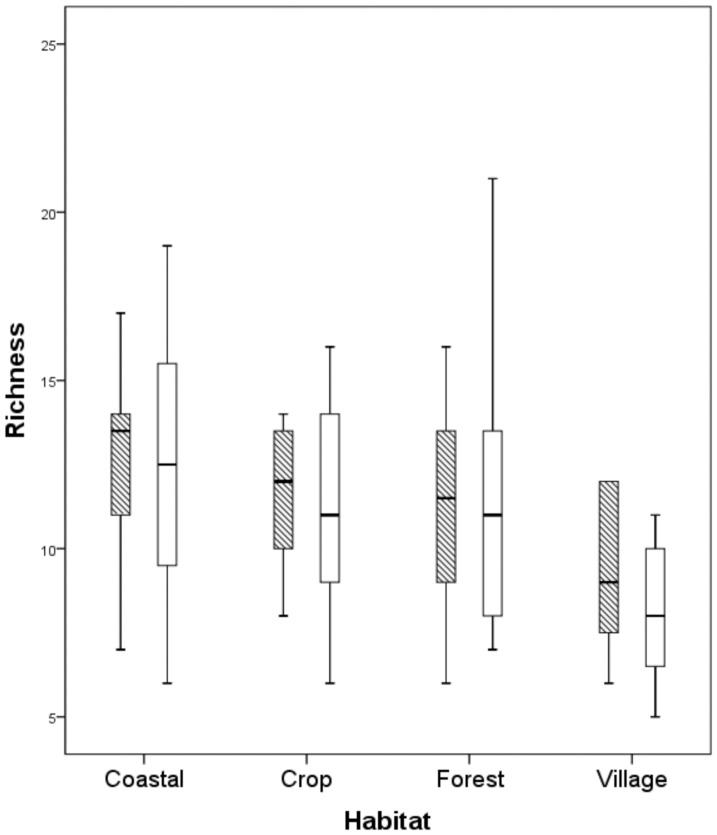
Boxplot showing species richness for each sampled habitat where mongoose were absent (shaded bars) or present (white bars).

Inspection of the rarefraction curves revealed that the village habitat had generally lower richness, and that mongoose-free islands tended to have higher bird abundance (Figure 5). Species richness was consistently slightly higher on mongoose-free islands across all habitat types (Figure 6). GLM revealed that for the village, crop and coastal habitats island quality influenced species richness (F = 11.35, P = 0.005, d.f. = 1,13; F = 6.8, P = 0.022, d.f. = 1,13; and F = 16.8, P = 0.001, d.f. = 1,13, respectively) but there was no measurable effect due to the presence of mongoose (F = 0.03, n.s., d.f. = 1,13; F = 0.67, n.s., d.f. = 1,13; and F = 2.7, n.s., d.f. = 1,13, respectively). For the forest habitat both island quality (F = 80.5, P<0.001, d.f. = 1, 13) and the presence of mongoose (F = 17.9, P<0.001, d.f. = 1, 13) influenced species richness.

**Figure 7:**
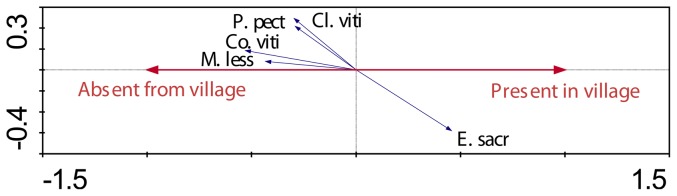
Species-environment biplot from pRDA summarizing differences in bird assemblages attributed to the presence of village habitat. Refer to Fig. 3 for more details.

**Figure 8:**
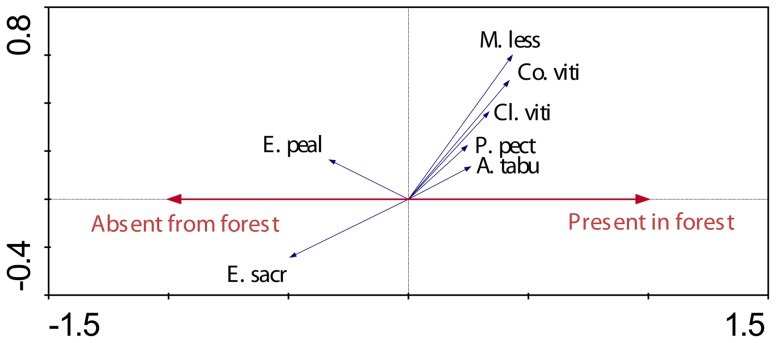
Species-environment biplot from pRDA summarizing differences in bird assemblages attributed to the presence of forest habitat. Refer to Fig. 3 for more details.

pRDA revealed that the presence of two habitat types (village and forest) had a measurable effect on species assemblage. The village habitat type explained 5.8% of variability in species assemblage (F = 4.576, P = 0.002). *P. pectoralis*, *Columba vitiensis*, *Cl. vitiensis* and *Myiagra vanikorensis* were strongly dissociated with this habitat (Figure 7) whilst *Egretta sacra* was associated. Similarly, the presence of forest habitat strongly influenced species assemblage, explaining 5.4% of variability (F = 4.278, P = 0.002). The species *Aplonis tabuensis, Co. vitiensis, Cl. vitiensis*, *M. lessoni* and *P. pectoralis* were strongly associated with this habitat type, with the opposite being the case for *Erythrura pealii* and *E. sacra* (Figure 8). It should be noted that *P. pectoralis*, *Co. vitiensis* and *Cl. vitiensis* were all dissociated with the village habitat and associated with the forest habitat respectively. No measurable effects of the scrub or crop habitats on species assemblage were detected.

## Discussion

Conjecture and anecdotal evidence have both played a part in attributing the decline of bird populations to the introduction of mongoose [53]–[56]. Extant bird communities are a result of the effects of decades of prior environmental modification as well as biogeographical factors including island size, isolation, and habitat complexity [15], [57]. Bird remains have been found in mongoose scats on the US Virgin Islands [26], on Hawai'i [58], [59], and on Korcula Island in the Adriatic [60], although studies conducted in Puerto Rico and Trinidad provided little evidence of bird predation [55], [61], [62]. It is also known that domestic poultry may constitute an important dietary component [59], [63]. Inevitably, studies that attempt to attribute bird declines to mongoose are constrained because effects are recorded *post hoc* following introduction, with little known of the initial avifauna prior to human impact. However, it is likely that when mongoose arrive on a ‘new’ island there is a rapid extirpation of the most vulnerable birds attributable to predation [32], [33]. In 1979, 30 mongoose were released on Amami-Oshima Island in Japan, increasing to 10,000 animals by 1999 with a consequent rapid loss of native birds and reptiles [64].

This study, by direct comparison of islands with and without mongoose, allowed us to investigate likely effects on bird communities. We conclude that habitat quality, and to a lesser extent the presence of mongoose, influence species assemblage on small islands in the Fiji group. Our study showed that three ground birds, *G. phillipensis*, *A. supericiliosa* and *P. porhyrio* were negatively influenced by the presence of mongoose as they were observed only on islands that were mongoose-free. *A. supericiliosa* eggs and chicks near the water's edge are often predated upon by mongoose [7] and presumably the same is true for the other two species. Other, less vulnerable species (such as small passerines) probably avoid the impact of predation due to behavioural factors. Few small passerine bird remains have been recorded in the diet of mongoose in Fiji from well-forested areas (presumably due to their canopy-based rather than ground-based behaviour); the bird remains found were mainly common introduced species [21].

Bird assemblages were strongly influenced by habitat quality. Our study indicated that *P. perousii, P. solitarius, C. victor*, *D. latrans, Cl. vitiensis, P. pectoralis, P. tabunesis*, and *F. carunculata* were more likely to be found on islands wherever reasonable tracts of forest remain (although even islands of the highest quality still had a large proportion of modified or secondary vegetation). These species are primarily frugivores, nectivores and insectivores requiring good canopy cover. Forest ecosystems require such species to provide services for the pollination and dispersal of plant propagules [65]; on some islands (Dravuni, Macuata-i-wai, Malake, Nananu-i-ra, and Yanuca) little natural vegetation remains intact and the islands are highly modified talasiga communities [43] which would severely limit the viability of such species.

On other islands (Beqa, Nananu-i-cake, Naviti, and Waya) secondary forest existed but it was dominated by introduced tree species whilst the other islands (Kioa, Koro, and Laucala) still had some good tracts of native forest. We only observed *P. pectoralis* and *Cl. vitiensis* in ‘true’ forest habitat and our results also indicated that *Co. vitiensis* was found more often in intact forest than other habitats as previously observed [7]. There are few islands in Fiji with greater than 50% of the original forest cover remaining [7] and our results suggest that protecting these natural forests as a priority would support the survival of Fiji's native avifauna.

The study also showed variation in species assemblage according to habitat – with village areas unsurprisingly being particularly poor habitats for Fiji's native and endemic species. Human activity and the consequent habitat alteration caused has provided an opportunity for many invasive species to proliferate in newly modified landscapes which in some cases has led them to become the dominant vertebrate. In this study, mongoose were particularly evident around village areas, due to discarded refuse and the high number of introduced birds. Mongoose have been tracked more frequently in high human-use areas in comparison to forested areas [66].

Two options are available to formally test the effect of mongoose predation on island birds. Firstly, mongoose could be introduced to islands and their impact monitored, but, this would clearly be ethically unacceptable. Secondly, mongoose could be eradicated from islands and the response observed; only then could the effect of mongoose rather than other human-induced changes be quantified in a definitive manner, although the presence of other introduced predators may confound such a study. Eradicating mongoose from islands has been suggested [67], but to date this has occurred on only six very small (<115 ha) islands [68], [69]. Currently, Japanese researchers are trapping mongoose on Okinawa and Amami-Oshima (between 2000 and 2009 24,136 mongoose were captured on Amami-Oshima alone) but already the cost exceeds US$14 million dollars and the programme is planned to continue until 2014 [69]. Nevertheless, with appropriate resources, planning, and the availability of much better kill traps [70] there are at least two islands in Fiji where mongoose should and could be eradicated, with the objective of conserving their exceptional bird diversity. These islands are Kioa (18.6 km^2^) and Yanuca Island (1.5 km^2^). Even though the habitat quality on Yanuca Island is relatively poor the potential to restore this island is high because it is near Suva, the capital of Fiji, and could provide significant ecotourism benefits such as those seen in New Zealand on Tiritiri Matangi Island in the Hauraki Gulf [71]. Toxic baits should also be considered, particularly if there are other predators on the islands [72]–[73]. Globally, there are already 64 islands around the world with mongoose [69] and so it would be useful to develop an effective strategy to prevent further island introductions as a precautionary measure. Currently, there are no internal regulations or mechanisms in Fiji preventing the introduction of mongoose from one island to another [74]. Although there are significant costs in the establishment of an internal biosecurity programme specifically targeting mongoose within Fiji, the likely negative impact of the introduction to islands like Taveuni, Kadavu and Koro would make this approach worthwhile [75]. It is also likely that other taxonomic groups are also being affected within the Fiji Islands. For example, it is probable that the herpetofauna is impacted by the presence of mongoose [76] and mongoose are known disease vectors in Fiji, Cuba and Grenada [63], [77], [78].

Even though predatory species contribute to the decline of island species, it is the preservation of habitat quality that is fundamental for the survival of Fiji's birds. Hence, both the management of invasive species [79] and the safeguarding of the structure and function of forests on these islands are needed. Furthermore, this must involve local community participation as this approach is the only one likely to succeed in the long term. Conserving quality habitat is a key factor when trying to preserve avifauna in Fiji, and elsewhere in the Pacific [7], [8], [43]. The development of community-led strategies to protect and enhance the remaining native forests could successfully preserve these important natural resources. Otherwise, it is likely that Fiji's endemic avifauna will ultimately perish and be replaced with a suite of exotic and ubiquitous invaders that thrive in heavily modified landscapes.

## Supporting Information

Table S1 **Observations of birds recorded on islands where mongoose were absent.** Numbers represent the count for each species at each station.
(DOC)

Table S2 **Observations of birds recorded on islands where mongoose were present.** Numbers represent the count for each species at each station.
(DOC)

## References

[1] BirdLife International (2008) State of the world's birds: indicators for our changing world. Cambridge: BirdLife International. 28 p.

[2] Holdaway RN (1999) Introduced predators and avifaunal extinction in New Zealand. In: MacPhee RDE, editor. Extinctions in near time: causes, contexts, and consequences. New York: Kluwer Academic/Plenum. pp. 189–238.

[3] Martin PS, Steadman DW (1999) Prehistoric extinctions on islands and continents. In: MacPhee RDE, editor. Extinctions in near time: causes, contexts, and consequences. New York: Kluwer Academic/Plenum. pp. 17–56.

[4] Atkinson IAE (2006) Introduced mammals in a new environment. In: Allen RB, Lee WG, editors. Biological Invasions in New Zealand. Ecological Studies 186. Berlin Heidelberg: Springer-Verlag. pp. 49–66.

[5] Veitch CR, Gaskin C, Baird K, Ismar SMH (2011) Changes in bird numbers on Raoul Island, Kermadec Islands, New Zealand, following the eradication of goats, rats and cats. In: Veitch CR, Clout MN, Towns DR, editors. Island invasives: eradication and management. Proceedings of the International Conference on Island Invasives, Gland, Switzerland. IUCN and Auckland: CBB. 542 pp.

[6] Ryan P (2000) Fiji's natural heritage. Auckland: Exisle Publishing. 287 p.

[7] Watling D (2001) A guide to the birds of Fiji and Western Polynesia; including American Samoa, Niue, Samoa, Tokelau, Tonga, Tuvalu and Wallis and Fortuna. Suva: Environmental Consultants (Fiji) Ltd. 272 p.

[8] Steadman DW (2006) Extinction and Biogeography of Tropical Pacific Birds. Chicago: The University of Chicago Press. 480 p.

[9] Swinnerton K, Maljkovic A (2006) Preliminary report on the status and distribution of the red-throated lorikeet (*Charmosyna amabilis*) in Fiji. Unpublished technical report submitted to the National Trust for Fiji, World Parrot Trust

[10] Worthy T (2000) The fossil megapodes (Aves: Medapodiiadae) of Fiji with descriptions of a new genus and two new species. Journal of the Royal Society of New Zealand 30: 337–364.

[11] Worthy T (2001) A new extinct species of snipe *Coenocorypha* from Viti Levu, Fiji. Bulletin of the British Ornithologists Club 123: 90–103.

[12] Worthy T (2004) The fossil rails (Aves: Rallidae) of Fiji with descriptions of a new genus and species. J R Soc N Z 34: 295–314.

[13] Atkinson IAE (1985) The spread of commensal species of *Rattus* to oceanic islands and their effects on island avifaunas. In: Moors PJ, editor. Conservation of island birds. ICBP Technical Publication 3: : 35–81.

[14] Blackburn TM, Gaston KJ (2005). Biological invasions and the loss of birds on islands. In: Sax DF, Stachowicz, JJ, Gaines SD, editors. Species Invasions: Insights into ecology, evolution and biogeography. Sunderland, Massachusetts: Sinauer Associates. pp. 85–110.

[15] Trevino HS, Skibeil AL, Karels TJ, Dobson FS (2007) Threats to avifauna on oceanic islands. Conserv Biol 21: 125–132. 17298518 10.1111/j.1523-1739.2006.00581.x

[16] Barun A, Simberloff D, Budinski I (2010) Impact of the small Indian mongoose (*Herpestes auropunctatus*) on native amphibians and reptiles of the Adriatic Islands, Croatia. Anim Conserv 13: 549–555.

[17] Pernetta JC, Watling D (1978) The introduced and native terrestrial vertebrates of Fiji. Pac Sci 32: 223–244.

[18] Simberloff D, Rejmánek M (2011) The Encyclopedia of Biological Invasions, University of California Press, 765 p.

[19] Veron G, Patou ML, Pothet G, Simberloff D, Jennings AP (2007) Systematic status and biogeography of the Javan and small Indian mongooses (Herpestidae, Carnivora). Zool Scr 36: 1–10.

[20] Patou ML, McLenachan PA, Morley CG, Couloux A, Cruaud C, et al (2009) Molecular phylogeny of the Herpestidae (Mammalia, Carnivora) with a special emphasis on the Asian Herpestes. Mol Phylogenet Evol 53: 69–80. 19520178 10.1016/j.ympev.2009.05.038

[21] Gorman ML (1975) The diet of feral *Herpestes auropunctatus* (Carnivora: Viverridae) in the Fijian Islands. J Zool 175: 273–278. 10.1111/j.1469-7998.1976.tb06010.x 955242

[22] Martin AH (1938) The Birds of Fiji. Transactions Fiji Society for Science and Industry 4–7.

[23] Mercer R (1970) A field guide to Fiji birds. Suva: Fiji Museum Special Publication Series No. 1.

[24] Clunie F, Morse P (1984) Birds of the Fiji bush. Suva: Fiji Museum.

[25] Baker JK, Russell CA (1979) Mongoose predation on a nesting nene. Elepaio 40: 51–52.

[26] Seaman GA (1952) The mongoose and Caribbean wildlife. Seventeenth North American Wildlife Conference 188–196.

[27] Seaman GA, Randall JE (1962) The mongoose as a predator in the Virgin Islands. J Mammal 43: 544–546.

[28] Cheke A (1987) An ecological history of Mauritius. In: Diamond AW, editor. Studies of Mascarene Island Birds. Cambridge: Cambridge University Press. pp. 5–89.

[29] Hays WST, Conant S (2003) Impact of the small Indian mongoose (*Herpestes javanicus*) (Carnivora: Herpestidae) on native vertebrate populations in areas of introduction. Pac Sci 61: 3–16.

[30] Williams CB (1918) The food habits of the mongoose in Trinidad. Bulletin of the Department of Agriculture, Trinidad and Tobago 17: 167–186.

[31] Allison NA (1980) Notes on birds of Viti Levu and Vanua Levu, Fiji. Sea Swallow 29: 10–11.

[32] Blackburn A (1971) Some notes on Fijian birds. Notornis 18: 147–174.

[33] Clunie F (1972) Fijian birds of prey. Fiji Museum Educational Series

[34] Gorman ML (1973) Habitats of the land-birds of Viti Levu, Fiji Islands. Ibis 117: 152–161.

[35] Holyoak DT (1977) Notes on the birds of Viti Levu and Taveuni. The Emu 79: 7–76.

[36] Tarburton MK (1992) Weights of some birds from Fiji. Bulletin of the British Ornithologists' Club 112: 34–38.

[37] Hartlaub G (1864) Provisional list of a collection of birds in the Feejee Islands. Ibis 232.

[38] Layard EL (1876) Notes on some little-known birds of the new colony of the Fiji Islands. Ibis 137–152.

[39] Finch O (1877) Reports on the collection of birds made during the voyage of HMS ‘Challenger’ No IV, on the birds of Tangatabu and Fiji islands, Api (New Hebrides), and Tahiti. Proceedings of the Zoological Society of London 723–742.

[40] Bahr PH (1912) On a journey to the Fiji islands, with notes on the present status of their Avifauna, made during a year's stay in the group. Ibis 6: 282–314.

[41] Wood C, Wetmore A (1925) A collection of birds from the Fiji Islands. Ibis 1: 814–855.

[42] Watling D (1985) Notes on the birds of Gau Island, Fiji. Bulletin of the British Ornithologists' Club 105: 96–102.

[43] Franklin J, Steadman DW (2010) Forest plant and bird communities in the Lau Group, Fiji. PlosOne 5: 1–14. 10.1371/journal.pone.0015685 PMC3012085 21206753

[44] Beckon W (1988) Distribution of land birds in Fiji. Unpublished Report. University of California, Davis 152.

[45] Strafford JMC, Rodda P (2000) Late Miocene to Pliocene palaeogeography of Viti Levu, Fiji Islands, Palaeogeogr, Palaeoclimataol, Palaeoecol. 162: 137–153.

[46] SPREP (2003) Action strategy for nature conservation in the Pacific Islands Region, 2003–2007. South Pacific Regional Environment Programme, Apia, Samoa.

[47] Thaman RR, Keppel G, Watling D, Thaman B, Gaunavinaka T, et al (2005) Nasoata mangrove island, the PABITRA coastal study site for Viti Levi, Fiji Islands. Pac Sci 59: 193–204.

[48] Gotelli NJ, Colwell RK (2001) Quantifying biodiversity: procedures and pitfalls in the measurement and comparison of species richness. Ecol Lett 4: 379–391.

[49] Leps J, Smilauer P (2003) Multivariate Analysis of Ecological Data Using CANOCO. Cambridge: Cambridge University Press. 269 p.

[50] ter Braak CJF, Smilauer P (2002) CANOCO Reference Manual and CanoDraw for Windows User's Guide. Wageningen: Biometris. 500 p.

[51] Hurlbert SH (1971) The non-concept of species diversity: a critique and alternative parameters. Ecology 52: 577–586. 28973811 10.2307/1934145

[52] Magurran AE (2004) Measuring Biological Diversity. Oxford: Blackwell Publishing. 256 p.

[53] Allen GM (1911) Mammals of the West Indies. Bulletin of the Museum of Comparative Zoology, Harvard College 54: 175–263.

[54] King WB, Gould PJ (1967) The status of Newell's race of the Manx shearwater. Living Bird 6: 163–186.

[55] Stone CP, Dusek M, Aeder M (1994) Use of the anticoagulant to control mongooses in nene breeding habitat. Elepaio 54: 73–78.

[56] Vilella FJ, Zwank P (1993) Ecology of the small Indian Mongoose in a coastal dry forest of Puerto Rico where sympatric with the Puerto Rican nightjar. Caribb J Sci 29: 24–29.

[57] MacArthur RH, Wilson EO (1967) The Theory of Island Biogeography. Princeton: Princeton University Press. 203 p.

[58] Baldwin P, Schwartz CW, Schwartz ER (1952) Life history and economic status of the mongoose in Hawai'i. J Mammal 33: 335–355.

[59] Kami HT (1964) Foods of the mongoose in the Hamakua District, Hawai'i. Zoological Record 3: 165–170. 5895902

[60] Cavallini P, Serafini P (1995) Winter diet of the small Indian mongoose *Herpestes auropunctatus*, on an Adriatic Island. J Mammal 76: 569–574.

[61] Pimental D (1955) Biology of the Indian mongoose in Puerto Rico. J Mammal 36: 62–68.

[62] Urich FW (1931) The mongoose in Trinidad. Tropical Agriculture 8: 95–97.

[63] Nellis DW, Everard COR (1983) The biology of the mongoose in the Caribbean. Foundation for Scientific Research in Surinam and the Netherlands Antilles.

[64] Yamada F (2002) Impacts and control of introduced small Indian mongoose on Amami Island, Japan. In: Veitch, C.R. and Clout, M.N. (eds.). Turning the tide: the eradication of invasive species. Proceedings of the International Conference on Eradication of Island Invasives. IUCN, Auckland, New Zealand. pp. 389–392.

[65] Steadman DW, Freifeld HB (1999) The food habits of Polynesian pigeons and doves: a systematic and biogeographic review. Ecotropica 5: 13–33.

[66] Quinn J, Whisson DA (2005) The effects of anthropogenic food on the spatial behaviour of small Indian mongooses (*Herpestes javanicus*) in a subtropical rainforest. J Zool 267: 339–350.

[67] Roy SS, Jones CG, Harris S (2002) An ecological basis for control of the mongoose *Herpestes javanicus* in Mauritius: is eradication possible? In: Veitch CR, Clout, M, editors. Turning the tide: the eradication of invasive species. Proceedings of the International Conference on Eradication of Island Invasives. IUCN, Auckland, New Zealand. pp. 266–273.

[68] Lorvelec O, Delloue X, Pascal M, Mège S (2004) Impacts des mammifères allochtones sur quelques espèces autochtones de l'îlet Fajou (Rèerve naturelle du grand cul-de-sac Marin, Guadaloupe), Établis ÀL'issue D'une tentative d'eradication. Revue d'Ecologie 59: 293–307.

[69] Barun A, Hanson CC, Campbell KJ, Simberloff D (2011) A review of small Indian mongoose management and eradications on islands. In: Veitch CR, Clout MN, Towns DR, editors. Island invasives: eradication and management. Proceedings of the International Conference on Island Invasives, Gland, Switzerland. IUCN and Auckland: CBB. 542 p.

[70] Peters D, Wilson L, Mosher S, Rohrer J, Hanley J, et al. (2011) Small Indian mongoose – management and eradication using DOC 250 kill traps, first lessons from Hawaii. In: Veitch CR, Clout MN, Towns DR, editors. Island invasives: eradication and management. Proceedings of the International Conference on Island Invasives, Gland, Switzerland. IUCN and Auckland: CBB. 542 pp.

[71] Rimmer A (2009) Tiritiri Matangi: A model for conservation. New Zealand: Random House. 152 p.

[72] Griffiths R (2011) Targeting multiple species – a more efficient approach to pest eradication. In: Veitch CR, Clout, M, editors. Turning the tide: the eradication of invasive species. Proceedings of the International Conference on Eradication of Island Invasives. IUCN, Auckland, New Zealand. 542 p.

[73] Innes J, Saunders A (2011) Eradicating multiple pests: an overview. In: Veitch CR, Clout M, editors. Turning the tide: the eradication of invasive species. Proceedings of the International Conference on Eradication of Island Invasives. IUCN, Auckland, New Zealand. 542 p.

[74] Morley CG (2004) Actions speak louder than words: a call for preventing further mongoose invasions in Fiji. In: Timm RM, Gorenzel WP, editors. Proceedings of the 21st Vertebrate Pest Conference, Visalia, California. University of California. pp. 37–41.

[75] Morley CG (2004) Has the invasive mongoose *Herpestes javanicus* yet reached the island of Taveuni, Fiji? Oryx 38: 457–460.

[76] Hedges SB, Conn CE (2012) A new skink fauna from Caribbean islands (Squamata, Mabuyidae, Mabuyinae). Zootaxa 3288: 1–244.

[77] Nellis DW, Everard CO (1983) The biology of the mongoose in the Caribbean Islands. Studies on the fauna of Curacao and other Caribbean Islands 64: 1–162.

[78] Collins DF (1984) *Leptospira interrogans* infection in domestic and wild animals in Fiji. New Zealand Veterinary Journal 32: 21–24. 16031033 10.1080/00480169.1984.35050

[79] Simberloff D (2003) How much information on population biology is needed to manage introduced species? Conserv Biol 17: 83–92.

