# Supplementary material for: The Effect of the Small Indian Mongoose (Urva auropunctatus), Island Quality and Habitat on the Distribution of Native and Endemic Birds on Small Islands within Fiji
Source: PLoS One. 2013 Jan 17;8(1):e53842. doi: 10.1371/journal.pone.0053842 (PMC3547964; doi:10.1371/journal.pone.0053842)
Supplement: Table S1 — Observations of birds recorded on islands where mongoose were absent. Numbers represent the count for each species at each station. (DOC) [file pone.0053842.s001.doc]

Table S1. Observations of birds recorded on islands where mongoose were absent. Numbers represent the count for each species at each station.
